# 

**Published:** 1784

**Authors:** 


### THE LONDON MEDICAL JOURNAL.

### VOLUME THE FIFTH,

FOR THE YEAR 1784.

63444
S.G.O.
LIBRARY.

LONDON:

PRINTED for the EDITOR,

And sold by JOSEPH JOHNSON, LONDON;

CHARLES ELLIOT, EDINBURGH;

And P. BYRNE, DUBLIN

M,DCC,LXXXV.

### P R O M O T E D.

Lately, Dr. Anton. Scarpa of Modena, to be profeſſor of anatomy and ſurgery at Pavia.—M. Daubenton and M. Brouſſonet, to be profeſſors of rural œconomy in the veterinary ſchool at Paris.—M. Fourcroy, to be profeſſor of chemiſtry in the king's garden at Paris, in the room of the late M. Macquer.

1783.

1783. *Dec.* 27. John Philips, Efq. to be furgeon to the prince of Wales's houfhold.

1784. *Jan.* 8. Dr. Robert Hamilton, phyfician at Ipfwich, to be an extra-licentiate of the College of Phyficians, London.—12. Archibald Richardfon, Efq. ftate furgeon, to be the furgeon general in Ireland, in the room of William Ruxton, Efq. deceafed, and George Stewart, Efq. to be ftate furgeon in Ireland, in the room of Mr. Richardfon.—16. Dr. George Sandiman, to be phyfician to the General Difpenfary in London, in the room of Dr. Adair Crawford, who refigned.—17. Mr. James Barton, late of the 88th regiment of foot, to be furgeon to the 10th regiment of dragoons.—22. Mr. Edmund Pitts to be furgeon to St. Bartholomew's Hofpital, in the room of Mr. Robert Young, deceafed. – Mr. William Long to be affiftant furgeon to the fame hofpital, in the room of Mr. Pitts.

*Feb.* 26. Dr. William Hamilton, to be phyfician to the Surry Difpenfary, in the room of Dr. John Sims, who refigned.

*March* 4. Dr. William Pitcairne, to be treafurer of St. Bartholomew's Hofpital, in the room of John Darker, Efq. deceafed.—20. Mr. Ben-

 jamin jamin Shield, hoſpital mate, to be ſurgeon of the 58th regiment of foot.

---

### D E A T H S.

Lately at Dublin, William Ruxton, Eſq. ſurgeon general in Ireland.—At Enfield, Mr. Tho. Pritchard, ſurgeon.

At Paris, Mr. Ant. Franc. Barbaut, ſurgeon to the Chatelet, formerly profeſſor of midwifery in the Royal Academy of Surgery, and author of the following works; viz. 1. *Splanchnologie, ſuivie de l'angeiologie, et de la neurologie*, 12mo, Paris, 1739. 2. *Principes dc la chirurgie*, ibid. 12mo. 3. *Traité des accouchemens*, ibid. 1775, 2 vol.

1783. *Jan.* 16. At Straſburgh, Dr. John Pfeffinger, profeſſor of phyſic.

*Feb.* 12. At Farnham in Surry, aged 59 years, Mr. Richard Sumner, apothecary.

*April* 3. At Zutphen in Guelderland, aged 60 years, David de Gorter, M. D. formerly profeſſor of botany at Harderwyck, and for ſome time (viz. from 1754 till 1764) firſt phyſician to the empreſs of Ruſſia, a ſtation his father, ther, John de Gorter, had likewiſe the honour of filling. He was the author of ſeveral botanic works.

*Sept.* 8. At Straſburgh, John Reinbold Spielman, M. D. profeſſor of chemiſtry, botany, and the materia medica in that univerſity, and member of the academies of Peterſburg, Berlin, &c.; born at Straſburgh, March 31, 1722.

*Dec.* 20. At Morpeth, in Northumberland, aged 62 years, John Fenwick, M. D.—25. At Haverhill, in Cambridgeſhire, Mr. Hempſtead, apothecary.—30. In Great Ruſſel-ſtreet, Bloomſbury, Mr. Robert Young, ſurgeon of St. Bartholomew's Hoſpital, and one of the court of examiners of the company of ſurgeons, London.

1784. *Jan.* At Paris, M. Simon Claude Delaizé, jun. ſurgeon.——4. At Limehouſe, Mr. Minſon Hales, ſurgeon.

*Feb.* Loſt on board the packet from India, on the rocks of Scilly, Mr. William Perry, late ſurgeon of his majeſty's ſhip the Superb.—At Paris, Mr. Nicolas Simeon Caignard, and Mr. Henry Didier, ſurgeons. — 2. George Bell, M. D. Phyſician to the Infirmary and Lunatic Hoſpital at Mancheſter. He was a native of Dumfries, and graduated at Edin-

Edinburgh in 1780.—3. At Bradford in Yorkshire, aged 50 years, Mr. William Wright, apothecary. — 4. Mr. David Story, aſſiſtant ſurgeon to Greenwich Hoſpital.—5. At Enfield, Mr. Thomas Brown, ſurgeon, late of Peckham in Surry.—At Kentiſh Town, Mr. John Freake, apothecary. —13. At Stroud in Glouceſterſhire, Samuel Jones, M. D. — 17. At Paris, Peter Joſeph Macquer, M. D. a celebrated chemical writer, of whoſe life we mean hereafter to give ſome account.—26. At Mancheſter, Samuel Kay, M. D. formerly phyſician to the Infirmary of that place. He was a native of Lancaſhire, and graduated at Edinburgh in 1731.—27. At Skelton in Cleveland, Mr. Thomas Busfield. formerly an apothecary at York.

*March.* At Preſton in Lancaſhire, Dr. Heyes. —7. At Canterbury in Kent, aged 75 years, Hugh van der Poll, M. D. in the univerſity of Leyden, where he graduated in 1743, and formerly in great repute as a phyſician at Amſterdam. The public are indebted to him and Dr. Jacob Viſſcher, another phyſician at Amſterdam, for a deſcription of the lever invented by Roonhuyſen (ſee vol. iv. p. 162).—At Plymouth, M. Nicholas May, ſurgeon and apothecary.—24. At Kennington in Surry, Matthew Morley,

M. D.

M. D. in the univerfity of Cambridge, and fel- of the royal college of phyficians, London, into which he was admitted in 1740.

---

### SECTION VI.

### QUARTERLY CATALOGUE.

1. A Serious and friendly addrefs to the public on the dangerous inconveniencies of neglecting common coughs and colds, fo frequent in this climate, containing a fimple and efficacious method of cure. By a Gentleman of the Faculty. 8vo. *Murray*, 1784. 1s. 6d.

2. Curfory obfervations on a treatife intituled, "Medical advice to the confumptive people of England, by Philip Stern, M. D." Addreffed to the confumptive people of this kingdom. By *Thomas Hodfon*. 8vo. *Murray*, 1784. 1s.

3. Chemical reflections relating to the nature, caufes, prevention, and cure of fome difeafes; particularly the fea-fcurvy, the ftone and gravel, the gout, the rheumatifm, &c. Containing obfervations upon air; upon conftituent principles; and the decompofition of animal and vegetable fubftances: with a variety of occafional remarks, philophilosophical and medical. To which is added, the method of making wine from the juice of the sugar cane. By *James Rymer*, surgeon at Ryegate. 12mo. *Evans*, London, 1784. 202 pages.

4. The philosophy of physic; or phlogistic system, in which phlogiston supplied in an aerial form by the ingesta, and regulated in its agencies and evolutions by atmospherical and tonic re-actions, is considered as constituting, actuating, and supporting the vital power or stimulating susceptibility; and hence a concise plan of medical practice is proposed on fixed principles, which result from a general and particular view of causes and effects. By *T. Dewell*, surgeon. 8vo. *Murray*, 1784. 47 pages. 1s. 6d.

5. A system of anatomy from Monro, Winslow, Innes, and the latest authors; arranged, as nearly as the nature of the work would admit, in the order of the lectures delivered by the professor of anatomy in the university of Edinburgh. 8vo. 2 vols. *Elliot*, Edinb. 1784. With 16 copper-plates. Vol. I. p. 440. vol. II. p. 587.

6. Observations on Hepatic diseases incidental to Europeans in the East Indies. By *Stephen Matthews*, surgeon in the Honourable United East India Company's service and formerly of the the Duke of Portland Eaſt Indiaman. 8vo. *Cadell*, London, 1783. 4 s.

7. Diſſertations on ſelect ſubjects in chemiſtry and medicine. By *Martin Wall*, M. D. phyſician at Oxford, public reader of chemiſtry in that univerſity, and late fellow of New College. 8vo. *Cadell*, London, 1783. 3 s.

Theſe ingenious diſſertations are three in number. The firſt is on the ſtudy of chemiſtry; in the ſecond the author offers ſome conjectures concerning the origin and antiquity of the uſe of ſymbols in aſtronomy and chemiſtry; and the third contains obſervations on the diſeaſes prevalent in the South Sea Iſlands, with ſome remarks concerning the firſt appearance of the lues venerea in Europe.

8. An eſſay on the principles and manners of the medical profeſſion; with ſome occaſional remarks on the uſe and abuſe of medicine. By *J. Whitaker Newman*, member of the Corporation of Surgeons of London. 8vo. *Dodſley*, London, 1783. 1 s. 6 d.

9. Aphoriſms compoſed for a text to practical lectures on the conſtitution and diſeaſes of children. By *Andrew Wilſon*, M. D. fellow of the royal college of phyſicians, Edinburgh, and phyſician to the General Diſpenſary for the relief

 of of the infant poor. 12mo. *Murray*, London, 1783. 1 s.

10. Outlines of mineralogy. Tranflated from the original of Sir Torbern Bergman. By *William Withering*, M. D. member of the royal medical fociety at Edinburgh. 8vo. *Robinfon*, London, 1783. 128 pages. 2 s. 6 d.

11. An effay on the moft efficacious means of treating ulcerated legs; in which the topical applications in general ufe are confidered, and fome new methods for relief propofed; with particular obfervations on the fafety of healing old ulcers. 8vo. *Nicholl*, London, 1783. 1s.

12. A fovereign Remedy for the Dropfy, publifhed by defire for public benefit. 4to. *Dodfley*, London, 1783. 11 pages, 6d.

The remedy here recommended confifts of a drachm of broom feed well powdered and fifted, which is to be infufed twelve hours in a glafs and a half of rich white wine, and taken in the morning fafting. The patient is afterwards to walk or ride for an hour and a half, and then to fwallow two ounces of olive oil. This method is to be repeated every other day, or once in three days, till the cure is compleated.—A fimilar prefcription may be feen in Lemery's *Recueil des fecrets*, tome 1, p. 192.

13.

13. A Syſtem of Vegetables according to their claſſes, orders, genera, and ſpecies, with their characters and differences, in 2 volumes. Tranſlated from the 13th edition (as publiſhed by Dr. Murray) of the *Syſtema vegetabilium* of the late profeſſor Linneus; and from the *Supplementum plantarum* of the preſent profeſſor Linnæus. By a Botanical Society at Litchfield. Vol. I. 8vo. *Leigh* and *Sotheby*, London, 1783. 424 pages, with 11 copper-plates.

This tranſlation, which is dedicated to Sir Joſeph Banks, Bart. is literal and accurate. The editors have been aſſiſted by their learned townſman Dr. Samuel Johnſon in forming a botanic language, of which a gloſſary is prefixed to the work. The technical words they have adopted are ſuch as would neceſſarily be uſed in an Engliſh converſation by botaniſts acquainted with the original of Linnæus, ſuch as *calyx*, *corol*, *piſtil*, *pericarp*, &c. and the diminutives of theſe, as *calycle*, *corollet*, &c. In a liſt of lovers of botany, to whom they return thanks for advice and aſſiſtance, we find enumerated "the learned and "ingenious ladies Mrs. Egerton of Oulton Park, "Cheſhire; Mrs. Blackburne of Orford in Lan-"caſhire; and Mrs. Cummins of Kenſington."

In an advertifement to the volume we are told that the editors intend as foon as poffible to publifh a tranflation of the *Genera* and *Species plantarum*, and have therefore only given the effential characters and fpecific diftinctions of plants in this work; intending to put the references and obfervations into their proper places in the Species, and the natural characters from the fupplement into the Genera.

14. Hortus Uptonienfis; or a Catalogue of ftove and green-houfe plants in Dr. Fothergill's garden at Upton at the time of his deceafe. 8vo. *Dilly*, London, 1783. 1s. 6d.

15. Defcription of a Glafs Apparatus for making in a few minutes, and at a very fmall expence, the beft mineral waters of Pyrmont, Spa, Seltzer, Seydfchutz, Aix la Chapelle, &c. together with the defcription of two new eudiometers, or inftruments for afcertaining the wholefomenefs of refpirable air, and the method of ufing thefe inftruments. In a letter to the Rev. J. Prieftley, L. L. D. F. R. S. By *J. H. de Magellan*, F. R. S. The 3d edition, revifed, corrected, and enlarged by the author, with an examination of the ftrictures of Mr. T. Cavallo, F. R. S. upon thefe eudiometers. 8vo. London, 1783. 80 pages.

16. Obſervations Medico-chemiques ſur le Cancer. *i. e.* Medico-chemical obſervations on Cancer. By *M. Martinet*, curate of Soulaines near Bar-ſur-Aubes. 8vo. Paris, 1781. 39 pages.

This work contains a great deal of erroneous theory. Its object is to recommend the external uſe of a ſolution of volatile alkali fluor in water in cancerous ulcers. Four caſes are related in which this application is ſaid to have been of uſe.

17. *Joſephi Jacobi Plenk* chirurgiæ doctoris, nec non chirurgiæ, anatomes atque artis obſtetriciæ profeſſoris in regia univerſitate Budenſi, &c. Pharmacologia chirurgica, ſive doctrina de medicamentis, quæ ad curationem morborum externorum adhibendi ſolent. 8vo. Vienna, 1783.

18. *Adalberti Vincentii Zarda*, philoſ. & med. doct. pharmaca vegetabilia juxta pharmacopeiam Auſtriaco provinc. 8vo. Prague, 1783. 364 pag.

19. Flore de Bourgogne; ou Catalogue des plantes naturelles à cette province, et de celles qu'on y cultive le plus communement, avec l'indication du ſol où elles croiſſent, du tems de leur fleuraiſon, et de la couleur de leur fleurs. *i. e.* A Burgundy Flora, or Catalogue of the indigenous plants of that province, and of thoſe which which are the moſt commonly cultivated there, with an account of the ſoil in which they grow, the time of their flowering, and the colour of their flowers. 2 vols. 8vo. Dijon, 1782.

This work is the production of M. Durande. The number of plants deſcribed in it amounts to 1284. The deſcription of theſe fills the firſt volume. In the ſecond volume he treats of their properties and uſes in phyſic, the arts, rural œconomy, &c.

20. Diſſertatio Chemica de Terra Abſeſtina quam preſide Mag. Torb. Bergman, chemiæ prof. reg. et ordin. nec non equite aurato reg. ord. de Waſa publicè ventilandam ſiſtit *Carolus Guſt. Robſahm*, Vermelandus. 4to. Upſaliæ, 1782. p. 16.

21. Diſſertatio Chemica de analyſi lithomargæ quam preſide Mag. Torb. Bergman, chemiæ prof. &c. publicè ventilandam ſiſtit ſtipendarius Gyllenjilmianus *Carolus Dieteric. Hjerta*, Weſtrogothus. 4to. Upſaliæ, 1782. p. 14.

22. Diſſertatio Gradualis ſiſtens chemiæ progreſſus a medio ſæc. vii. ad medium ſæc. xvii. cujus partem priorem preſide Mag. Torb. Bergman, chemiæ prof. &c. publicè ventilandam exhibet *Petrus Afzelius Arvidſon*, Weſtrogothus. 4to. Upſaliæ, 1782. p. 40.

23.

23. Elogio del Padre Beccaria, chierico regulare delle Scuole Pie, professore di fisica sperimentale nella regia universita di Torino delle acc. di Londra e di Bologna, membro onorario della R. accademia di pittura e scultura di Torino. 12mo. Torino, 1781. p. 30.

This eulogium, which is elegantly printed on vellum, was pronounced by Count Tana, Nov. 8, 1781, at the academy of painting at Turin. It affords but an imperfect view of the history and writings of the learned father Beccaria. We here learn, that he was born Oct. 2, 1716, and died after a painful and lingering illness, May 27, 1781. The English translation of his works was the book he opened with the most pleasure. He was a great admirer of Virgil, Catullus, and Dante, and is said to have had a taste for pictures.

24. Aanmerkingen over de Veranderingen, walke de Steenen in de Pisblaas der menschen ondergaan.—Brief over het Steensnyden in twee reisen volgens P. Franco.—Verhandeling van den Heere Maret, over de Voordeelen van het steensnyden in twee tyden.—Als mede de Stelrigels van Celsus, Albucasis en Le Dran, over deese Konstbewerking.—Gestaafd door de Waarnemingen van de heeren Ten Haaf en Van Wy opopgesteld, overgezet, en met Verklaaringen witgegeeven door *Petrus Camper.* i. e. Observations on the changes which the stone in the human bladder undergoes.— A letter concerning the operation of lithotomy at two different times as recommended by P. Franco, and an essay by M. Maret on the advantages of lithotomy performed in this manner, together with the rules laid down by Celsus, Albucasis, and Le Dran on this subject, and the results of the experiments of Messieurs Ten Haaf and Van Wy. Written, translated, and illustrated with notes, by *Peter Camper.* 8vo. Amsterdam, 1782. 203 pages.

25. Beytrage zur fieberlehre. *i. e.* Additions to the theory of fevers. By *Christian Frederic Elsner*, M. D. 8vo. Koningsberg, 1782. 142 pages.

26. Hebammenunterricht in Gesprachen, &c. *i. e.* Obstetric institutes for the use of pregnant and lying-in women. By *John Christopher Stackens*, professor of physic at Jena. 8vo. Jena, 1783. 216 pages.

